# Structure and Function in Homodimeric Enzymes: Simulations of Cooperative and Independent Functional Motions

**DOI:** 10.1371/journal.pone.0133372

**Published:** 2015-08-04

**Authors:** Stephen A. Wells, Marc W. van der Kamp, John D. McGeagh, Adrian J. Mulholland

**Affiliations:** 1 Department of Chemistry, University of Bath, Bath, United Kingdom; 2 Centre for Computational Chemistry, School of Chemistry, University of Bristol, Bristol, United Kingdom; Instituto de Tecnologica Química e Biológica, UNL, PORTUGAL

## Abstract

Large-scale conformational change is a common feature in the catalytic cycles of enzymes. Many enzymes function as homodimers with active sites that contain elements from both chains. Symmetric and anti-symmetric cooperative motions in homodimers can potentially lead to correlated active site opening and/or closure, likely to be important for ligand binding and release. Here, we examine such motions in two different domain-swapped homodimeric enzymes: the DcpS scavenger decapping enzyme and citrate synthase. We use and compare two types of all-atom simulations: conventional molecular dynamics simulations to identify physically meaningful conformational ensembles, and rapid geometric simulations of flexible motion, biased along normal mode directions, to identify relevant motions encoded in the protein structure. The results indicate that the opening/closure motions are intrinsic features of both unliganded enzymes. In DcpS, conformational change is dominated by an anti-symmetric cooperative motion, causing one active site to close as the other opens; however a symmetric motion is also significant. In CS, we identify that both symmetric (suggested by crystallography) *and* asymmetric motions are features of the protein structure, and as a result the behaviour in solution is largely non-cooperative. The agreement between two modelling approaches using very different levels of theory indicates that the behaviours are indeed intrinsic to the protein structures. Geometric simulations correctly identify and explore large amplitudes of motion, while molecular dynamics simulations indicate the ranges of motion that are energetically feasible. Together, the simulation approaches are able to reveal unexpected functionally relevant motions, and highlight differences between enzymes.

## Introduction

Enzyme-catalysed reactions take place in the enzyme active site, which is often sequestered from bulk solvent (to avoid loss or breakdown of intermediates, or to prevent side reactions). Substrate binding, conversion to product and product release often therefore require closing and opening of the active site, which can involve significant conformational change. Many enzymes exist as homodimers, with two equivalent active sites. Forming a dimeric (rather than monomeric) species has an entropy cost, which must be offset by the enthalpy of binding between the subunits. Cooperativity between subunits leading to enhanced activity has been suggested as a driver for this adaptation. Here, we examine two homodimeric enzymes for which crystallography suggests cooperative motions related to ligand binding: the human scavenger decapping enzyme DcpS (EC 3.6.1.59) and mammalian (*S*)-citrate synthase (CS; EC 2.3.3.1).

**Fig 1:**
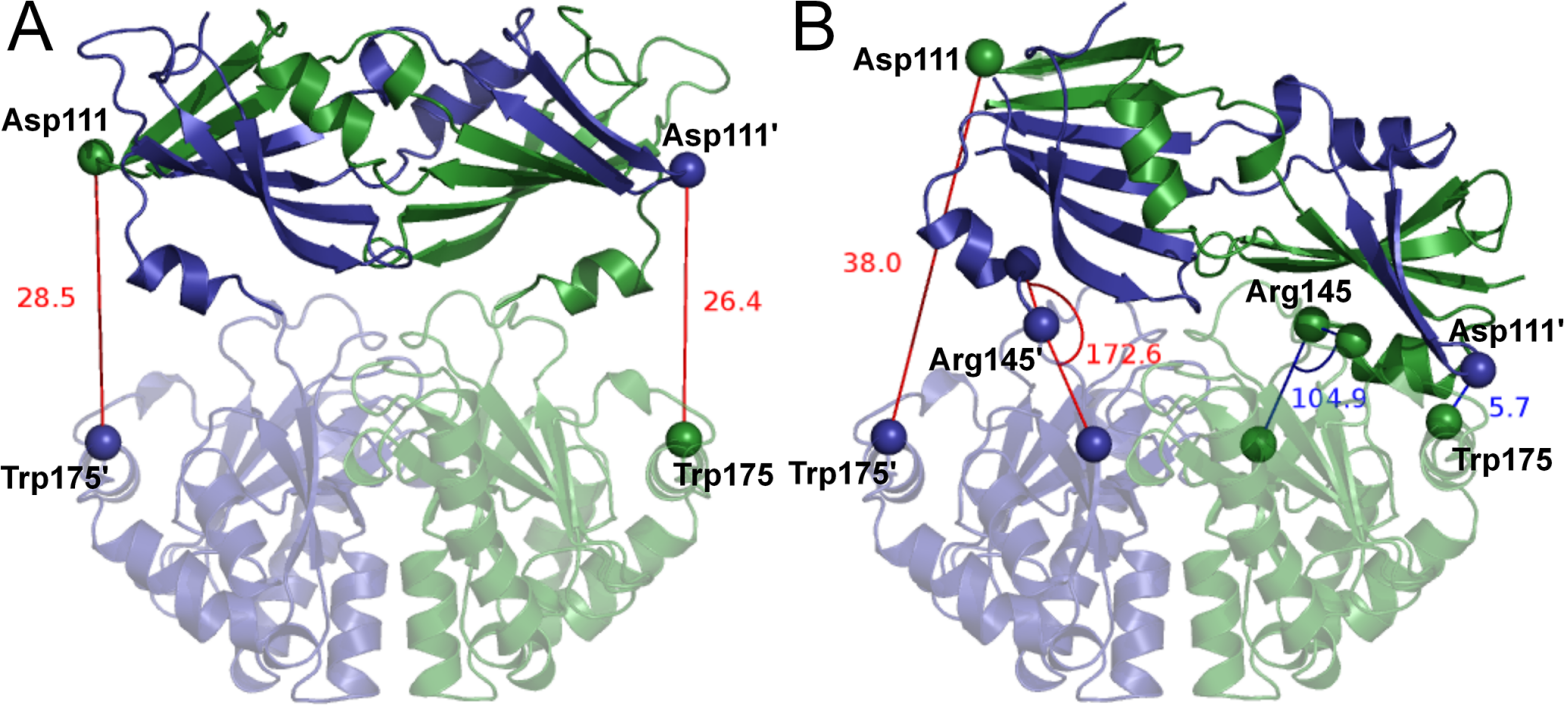
DcpS crystal structures with the cleft-distance and the ‘hinge-angle’ measurements labelled. Chain A is depicted in dark purple, chain B in dark green, with C-terminal domains transparent. A) symmetric structure, PDB ID: 1XML.[1] B) asymmetric structure, PDB ID: 1XMM (complexed with m7GDP; not shown here).[1]

DcpS is an enzyme that catalyses the hydrolysis of the 5’ cap structure in eukaryotic mRNA, a central step in mRNA turnover and regulation of gene expression. Crystal structures of this domain-swapped homodimeric enzyme indicate a symmetric apo-form with two open active sites (PDB ID: 1XML) and an asymmetric ligand-bound state (PDB IDs: 1XMM, 1ST0, 1ST4) in which one active site is closed with a ligand bound, and the other is open with no ligand bound [1, 2]. The active sites are located between the N- and C-terminal domains (with a domain-swapped N-terminal domain in the dimer, see Fig 1). Molecular dynamics (MD) simulations[3] revealed that in solution, the apo-form undergoes a continual process of conformational change, where one active site opens (by the N- and C-terminal domains moving away from each other) and the other closes (though not to the full extent of the bound crystal structure), in a strikingly anti-symmetric cooperative fashion: as one closes, the other opens. Flexible hinge regions connecting the N- and C-terminal domains permit this ‘rocking’ motion.

Citrate synthase catalyses the introduction of carbon into the citric acid cycle through the condensation of acetyl-CoA with oxaloacetate to form citrate. CS is found across all kingdoms of life with a well-conserved overall fold[4]. All known eukaryotic CS enzymes, as well as CSs from extremophiles, are homodimeric, with each subunit containing a large and a small domain. The main body of the dimer consists of the large domains from two subunits. Catalysis requires the binding of oxaloacetate and acetyl-CoA in the active site, which contains residues from both subunits. CS has become an archetype of enzyme conformational change.[5, 6] The many crystal structures available of citrate synthase indicate that a large conformational change occurs upon ligand binding (see e.g. [7]): the domains come together, causing the active site(s) to close and thereby be ready for catalysis. This open to closed change in CS has become an important model example of an enzyme conformational change, and a range of computational methods has been applied to investigate the transition pathway.[8–12] In all crystal structures, the two active sites are either both open (in the *apo* form, without ligands) or both closed. Previously, short (up to 2 ns) conventional atomistic MD[10] and principal component-based enhanced sampling (‘essential dynamics’) MD simulations[11] have been used to investigate the open/closed conformational change in CS. These authors concluded that there must be a significant energy barrier to reach the closed conformation in the absence of ligands, related to the interaction of α-helices 328–341 and 222–235 that form part of the ‘shear’ interface of the domain movement. Partly based on these data, Snow *et al*. later suggested[6] that the absence or presence of significant energy barriers between the open- and closed-domain conformations of unliganded enzymes is related to the mechanism of domain closure: they proposed that a ‘shear’ mechanism[5, 13] (as in CS) would lead to a large barrier, whereas a ‘hinge’ mechanism (as in DcpS[1]) would lead to an insignificant barrier, allowing the apo-enzyme to sample both open and closed states. Whether the open-to-closed (or vice versa) conformational change in CS occurs through a (symmetric) cooperative motion (both active sites closing at the same time) or through a non-cooperative motion (both active sites closing independently of each other), or both, is not yet known. A recent study has shown that normal modes of motion describing both symmetric and antisymmetric motions are a well-conserved feature of CS structures across the tree of life. [12]

Conformational changes can be directly related to the chemical changes that occur upon reaction, as we have suggested for CS[14]: the involvement of an arginine in the condensation reaction (forming citryl-CoA) may induce a closed-to-open conformational change (in the site where reaction takes place) that would allow for citryl-CoA hydrolysis. Kinetic cooperativity, i.e. substrate binding or reaction at one active site influencing catalysis of the reaction at another, however, has been excluded for dimeric CS.[15] For DcpS, it was suggested (based on the crystal structures) that binding of substrate to one site is coupled with reaction and product release in the other site.[1] Indeed, kinetic analysis of the DcpS reaction revealed negative cooperativity under high substrate concentrations,[16] consistent with the anti-symmetric cooperative motion observed in MD simulation.[3]

Here, we investigate the flexibility and conformational change in the unliganded structures of DcpS and CS using two different all-atom methods. The first approach is unbiased, all-atom molecular dynamics simulations (in explicit solvent) with a standard molecular mechanics empirical potential force-field approach. From the dynamic trajectories we extract principal components describing the overall motion, as well as local structural measured describing active-site geometries. The second approach is rapid geometric simulations of flexible motion. This approach combines constraint information from rigidity analysis using FIRST software[17] and directional information from coarse-grained elastic network analysis using Elnemo software[18] on the crystal structure to carry out template-based geometric simulations using the FRODA module within FIRST[19, 20], exploring large amplitudes of flexible motion. We stress that the geometric simulations do not merely project atoms linearly along normal mode directions; rather they maintain the local steric, covalent and noncovalent bonding geometry of the input structure while exploring large amplitudes of motion. The two methods give consistent results in the character of the motions of both enzymes. In the case of DcpS we find that the anti-symmetric cooperative functional motion suggested by crystallography, and a symmetric cooperative motion, are both intrinsic features of the protein structure. In the case of CS we find that symmetric and anti-symmetric motions are intrinsic to the protein structure; the MD trajectories of this enzyme explore combinations of both modes leading to an effectively independent, rather than cooperative, motion of the two binding clefts. These two homodimeric enzymes show distinct differences in their conformational behaviour, which is not apparent from the crystal structures alone. It seems likely that their specific conformational behaviour is related to their biological function; the dimeric nature of the proteins helps encode this behaviour.

## Results

### DcpS motion

The dimeric DcpS structure has a large C-terminal domain topped by a smaller N-terminal domain. Closure of the active site can be quantified by the approach of Asp111 located in a beta-hairpin on the N-terminal domain to Trp175 of the other subunit, located in a helix on the C-terminal domain (Fig 1); this measure has been used in the analysis of a previous MD investigation[3]. In the 1XML ‘doubly-open’ structure, the N-terminal domain sits mostly symmetrically atop the C-terminal domain and the Asp111-Trp175 inter-active-site distances are about 27 Å (Cα_Asp111_-Cα_Trp175’_ distance; denoted intersite distance from hereon) on both sides. In the closed 1XMM structure, the N-terminal domain has moved to an asymmetrical position so that Asp111 from chain B is adjacent to Trp175 from chain A, forming an active site which we term an "A-B" site. The closed Asp—Trp distance *d*(AB) in the 1XMM structure is 5.7 Å while the open d(BA) is 36.7 Å, illustrating the substantial scale of the domain motion. The structure should clearly be capable of forming an equivalent "B-A" site by closing in the opposite direction. We generate a model of such a site for comparison by forming a "swapped-1XMM" structure in which the chain identities A,B are simply reversed. Both flexible motion and MD simulations start from the 1XML apo-structure (both active sites open, see Fig 1).

Several structural descriptors may be used to describe the motion of the enzyme. As mentioned above, active site closure can be quantified by simple distance measurement, Asp111-Trp175 (Fig 1). We thus have a pair of ‘active-site-closure’ distances, *d*(AB) and *d*(BA). We may also consider a pair of angles which describe the conformation of the ‘hinge’-domain in both chains[3] (Fig 1B); this gives us a chain-A angle and a chain-B angle, with the functional motion involving a closing of one angle and an opening of the other. Finally, we may consider RMSDs relative to the open 1XML structure and to the closed 1XMM structure, or to the chain-swapped 1XMM structure if appropriate. The use of these measures allows us to discuss flexible motion and MD trajectories on a common basis. Additional validation of our distance measures by comparison to MD trajectory information is discussed below.

### Molecular dynamics simulations of DcpS

A previous molecular dynamics study of DcpS[3] reported on the behaviour of the structure during two 20ns simulation runs. This study showed a "strikingly cooperative" anti-symmetric motion, in which one active site closes while the other opens. The crystallographically observed near-symmetric apo structure was rarely populated (15–20% of the simulation time); rather, the structure passed briefly through the near-symmetric state several times in the course of its motion, but was usually asymmetric. The amplitudes of closing and opening motion were substantial (tens of Å), although the shortest active-site distance observed in the study, about 12 Å, is considerably greater than the 5.7 Å seen in the 1XMM closed structure. A very limited capacity for a symmetric closing motion was observed, but only when both intersite distances are large, with conformations showing simultaneous *d*(AB) and *d*(BA) distances of about 20 Å, compared to 27 Å in the input apo structure. The results of that study are overall fully consistent with the results of the flexible motion simulations.

**Fig 2:**
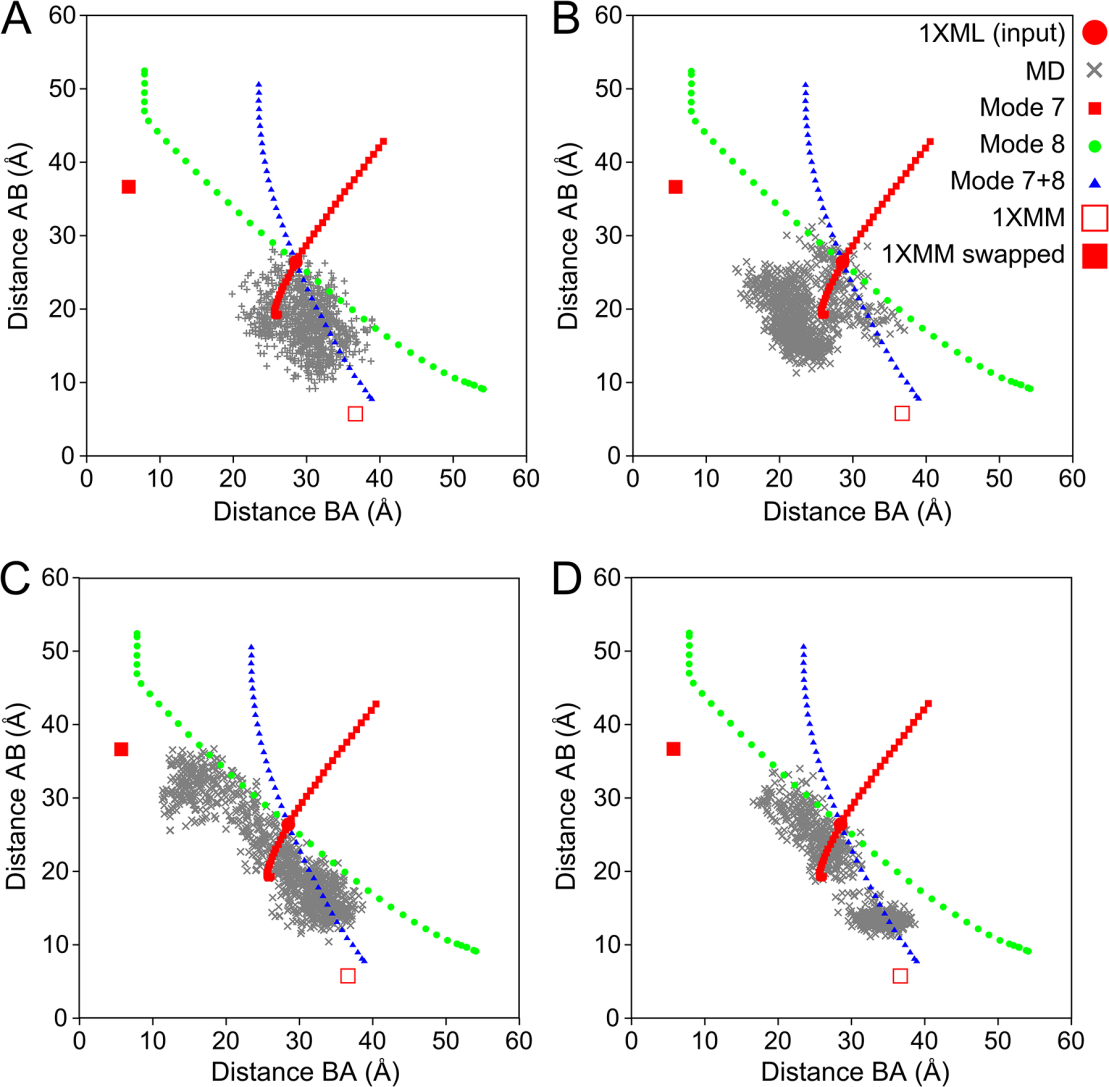
Comparison of geometric simulation and molecular dynamics trajectories of DcpS. 1XML refers to the (approximately) symmetric structure (see Fig 1A), 1XMM to the assymetric structure (see Fig 1B) and 1XMM swapped to the asymmetric structure with swapped chain IDs. Plots of the Cα distance between Trp175 and Asp111’ for both actives sites (“AB” and “BA”) as observed in the flexible motion trajectories biased along modes 7, 8 and the linear combination of 7+8 (small closed symbols) and in the individual MD trajectories (crosses for every 100 ps); A) run 1, B) run 2, C) run 3, D) run 4.

Here, we have extended our molecular dynamics investigation of DcpS to four independent trajectories of 100 ns each[21]. Fig 2 shows the range of intersite distances *d*(AB) and *d*(BA) explored during each of the MD runs (1–4), along with the ranges explored by the flexible motion simulations (see below). Different MD runs can display strikingly different behaviour in their exploration of the conformational space, with for example run 2 exploring mostly the symmetric closure motion while runs 1, 3 and 4 explore anti-symmetric motions in one direction (run 1) or both (runs 3,4). The extent of the motion during these longer MD trajectories naturally exceeds that explored in the previous study. The shortest active-site distance observed during asymmetric closure is *d*(AB) = 8.2 Å, while the shortest active-site distances achieved in a symmetric closure have *d*(AB) = *d*(BA)≈17 Å.

The correlation between *d*(AB) and *d*(BA) in the full 100 ns trajectories varies from near zero in run 2 to strongly negative (R<−0.9) in runs 3 and 4. This is consistent with a picture in which the motion of the domains is sometimes dominated by the anti-symmetric cooperative mode, and sometimes has components of both the anti-symmetric and symmetric modes, leading to effectively independent (non-cooperative) motion of the two active site clefts (but only when both clefts are largely open). The lack of symmetric motion in the MD simulations is evidenced by the fact that the highest correlation between *d*(AB) and *d*(BA) in any 10 ns window in the simulations is 0.3.

To validate the use of the d(AB)/d(BA) measures for describing the large-scale motion, we have compared these measures to the distance RMS of six-cross cleft distances (S4 Fig). Clustering of the MD trajectories (based on Cα RMSD of the N-terminal domains after alignment on the stable core of the C-terminal domains) further indicates that the single distance measures adequately represent the range of motion observed in the MD trajectories (Fig 2, S3 Fig). In addition to the intersite distances, Pentakainen et al.[3] quantified conformational changes in the “hinge” connecting the N- and C-terminal domains by measuring the angle formed between the Cα atoms of three residues: Tyr143, Arg145 (the centre of the hinge) and Arg149 (see Fig 1). We have also considered this hinge movement here, both for the flexible motion simulations and the MD simulations (S1 Fig). Flexible motion simulations achieve variations of the hinge angles of sufficient magnitude to match the crystallographically observed 1XML-1XMM transition. This involves an opening of one hinge by approximately 35 degrees with a closure of the other hinge by a similar amount. The MD simulations likewise achieve substantial variations in the hinge angles; however, it is clear that the hinge angles are highly susceptible to local variations in the chain conformation of the hinge region and thus provide a very "noisy" description of the motion of the structure.

### Flexible motion simulations of DcpS

Flexible motion simulations begin with the generation of normal mode eigenvectors describing potential low-frequency (flexible) motions. In DcpS, we find that the two lowest-frequency non-trivial modes, modes 7 and 8, describe collective motions of the entire N-terminal domain relative to the C-terminal domain. Higher-frequency modes are more localised, i.e. they do not describe large-scale domain motion. These two mode directions, and combinations thereof, are therefore used as biases during geometric simulation of flexible motion. This motion retains the bonding geometry and non-covalent interaction network of the input structure, so that the conformations generated are physically plausible. The influence of the constraint network converts a linear bias into a curving trajectory of motion[20], so that the simulations explore tilting of the N-terminal domain relative to the C-terminal domain. The constraint network also generates a natural limit to the amplitude of motion explored (see Methods); once the motion becomes incompatible with the constraints, due to stretching of covalent or noncovalent bonds or due to steric clashes, exploration ceases.

**Fig 3:**
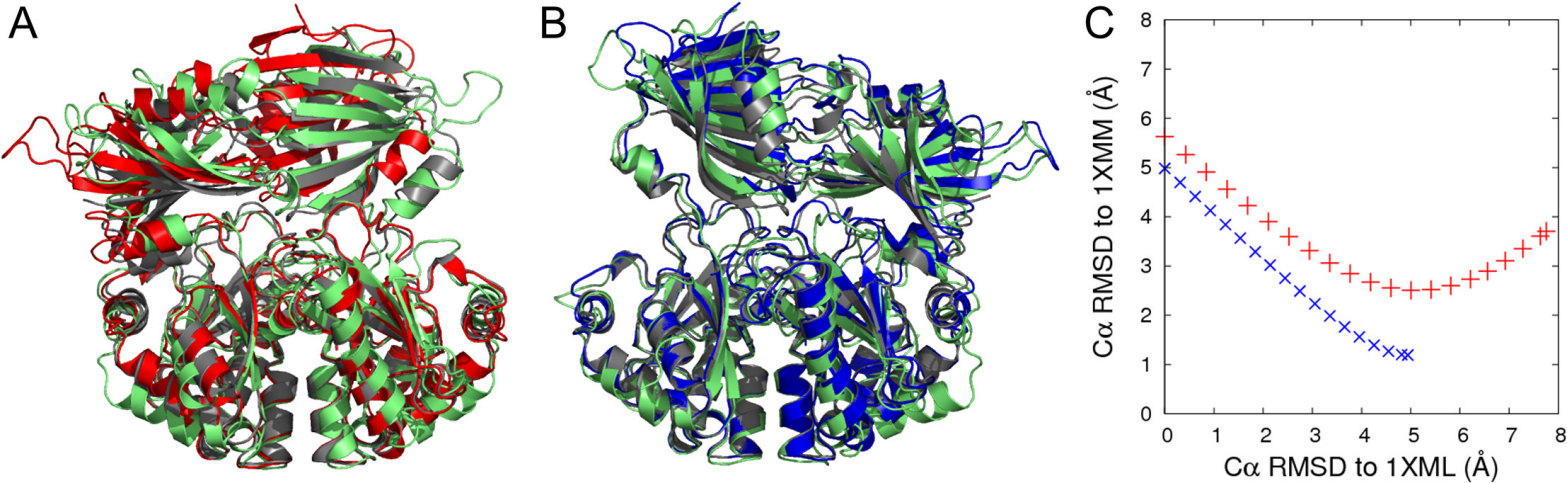
Structures obtained from geometric simulations of flexible motion biased along different normal mode directions (mode 7+8 in red–see text, mode 8 in blue) and MD simulations (light green) that are most similar to (A) the asymmetric structure (gray; PDB ID: 1XMM) and (B) the chain-swapped asymmetric structure (gray). C) Cα RMSDs to the symmetric (1XML) and asymmetric (1XMM) crystal structures for every 100^th^ frame of the flexible motion simulations along mode 8 (blue) and mode 8+7 (red).

On visual inspection of trajectories of flexible motion for 1XML, it is immediately apparent that motion biased parallel to mode 8 causes the N-terminal domain to tilt so that the active site closes (the beta hairpin carrying Asp111 approaches the helix carrying Trp175 on the C-terminal domain). This produces a structure quite similar to the 1XMM crystal structure; however, this flexible motion generates a "B-A" site rather than an "A-B" site, that is, the structure closes on the opposite side to that seen in the 1XMM crystal structure.[1] Fig 3B shows an overlay of a frame from this flexible motion with the "swapped-1XMM" structure; the Cα RMSD between the structures is 2.5 Å (see Fig 3C). This demonstrates that, firstly, the constraint network in the 1XML structure allows enough flexible motion for the structure to move directly to a 1XMM-like structure; and secondly, that the asymmetric cooperative motion involved in forming an active site is an intrinsic feature of the largely symmetric 1XML structure.

Due to the slight asymmetry of the starting structure, there is a consequent asymmetry in the normal modes; thus, motion biased antiparallel to mode 8 does not generate a truly 1XMM-like structure with an "A-B" site. Motion biased parallel or antiparallel to mode 7 does not generate appropriate motions of the N-terminal domain either. However, visual inspection of the motions suggested that a mode containing components of both modes 7 and 8 would generate an appropriate motion towards a 1XMM-like structure. We therefore constructed a composite mode containing equal components of modes 7 and 8; including normalisation, the resulting bias direction can be written as **e**_8+7_ = (1/sqrt(2))(**e**_8_ + **e**_7_). Motion biased antiparallel to this direction does generate a structure very similar to 1XMM (Fig 2A and 2C; Cα RMSD is 1.19 Å). This confirms that the 1XML structure has the intrinsic capacity for motion in both directions, to create either an "A-B" or "B-A" active site.

To quantify further the similarity between our simulated flexible motions and the crystallographic open/closed transition, we extract the inter site distances for the A-B and B-A sites. The closures achieved by flexible motion simulations give distances *d*(AB) = 7.9 Å in one case and *d*(BA) = 7.7 Å in the other; flexible motion has thus almost reached the degree of closure in the 1XMM crystal structure (*d* = 5.7 Å) starting from the input structure with *d*>27 Å.

Interestingly, bias along mode 7 on its own causes a more symmetric motion in which both the A-B and B-A active site distances increase or decrease simultaneously due to a "rolling" motion of the N-terminal domain over the C-terminal domain. The amplitude of this motion is however limited by the onset of steric clashes in the domain interface region, and so the two active sites cannot both close by motion along this mode. Exploration of this motion is only possible at large active-site distances; flexible motion in the closing direction jams at *d*(AB) = 19 Å, *d*(BA) = 26 Å.

### Overall character of motion in DcpS

Principal Component Analysis (PCA) of the MD trajectories shows that a first principal component (PC1), describing the rocking, "see-saw" anti-symmetric motion of the N terminal domain, accounts for 35% of the overall motion in the MD ensemble. A second component (PC2) describes a rotational motion of the N-terminal domain over the C-terminal domain and accounts for a further 16% of the overall motion. Motion along this PC2 component gives a correlated variation of the intersite distances whereas motion along PC1 gives anticorrelation. To transition from one asymmetric nearly-closed form to the other requires motions along both PC1 and PC2. S2 Fig shows the projection of the four MD trajectories onto the space of the principal comments PC1 and PC2, along with the location in this projection of the crystal structures. To provide an additional comparison between the flexible motion simulations and the MD essential dynamics, we also project the flexible motions biased along modes 7,8 and 8+7 onto the space of PC1/PC2. This projection shows that motion biased along mode 7 explores conformational space along PC2 over a range comparable to that explored by the MD trajectories, while motion biased along mode8 explores conformational space along PC1 over a range exceeding that explored by the MD trajectories. The difference in ordering of PCs 1 and 2 compared to modes 7 and 8 is not particularly significant since the exact ordering of normal modes can be altered by small changes to the distance cutoff or spring constants in the elastic network model. Thus the comparison of the methods in the space of PC1/PC2 is entirely consistent with the comparison in the space of the variables *d*(AB), *d*(BA).

We have assessed the convergence of our MD results by comparing the PC1/PC2 vectors generated over the full length of the MD trajectory (0-100ns) to those generated over three subsets (0-75ns, 0-85ns, 10-100ns). Generalised scalar products among these vectors (see S1 Table) show that the PCs generated over 0-100ns and over 10-100ns are effectively identical. The PC1,2 obtained over 0-75ns and 0-85ns windows span the same space as the full-length PC1,2, representing a slightly rotated form of the same basis set. The projections of the flexible motion trajectories onto these various PCs are shown in S6 Fig. Cosine contents of the full-length PCs are small, further indicating that PCs are not reflecting random fluctuations or diffusion (Cosine contents are 0.117 for PC1 and 0.002 for PC2 and less than 0.1 for other PCs.)Thus, the motions identified as principal components in the MD trajectories share the character of the motions explored by geometric simulations of flexible motion; in both cases we see a mode describing large amplitudes of anti-symmetric cooperative motion and a mode describing smaller amplitudes of symmetric cooperative motion, with components of both being required to describe the full transition from a structure closed on one side through the symmetric state to a structure closed on the opposite side.

### Citrate synthase motion

**Fig 4:**
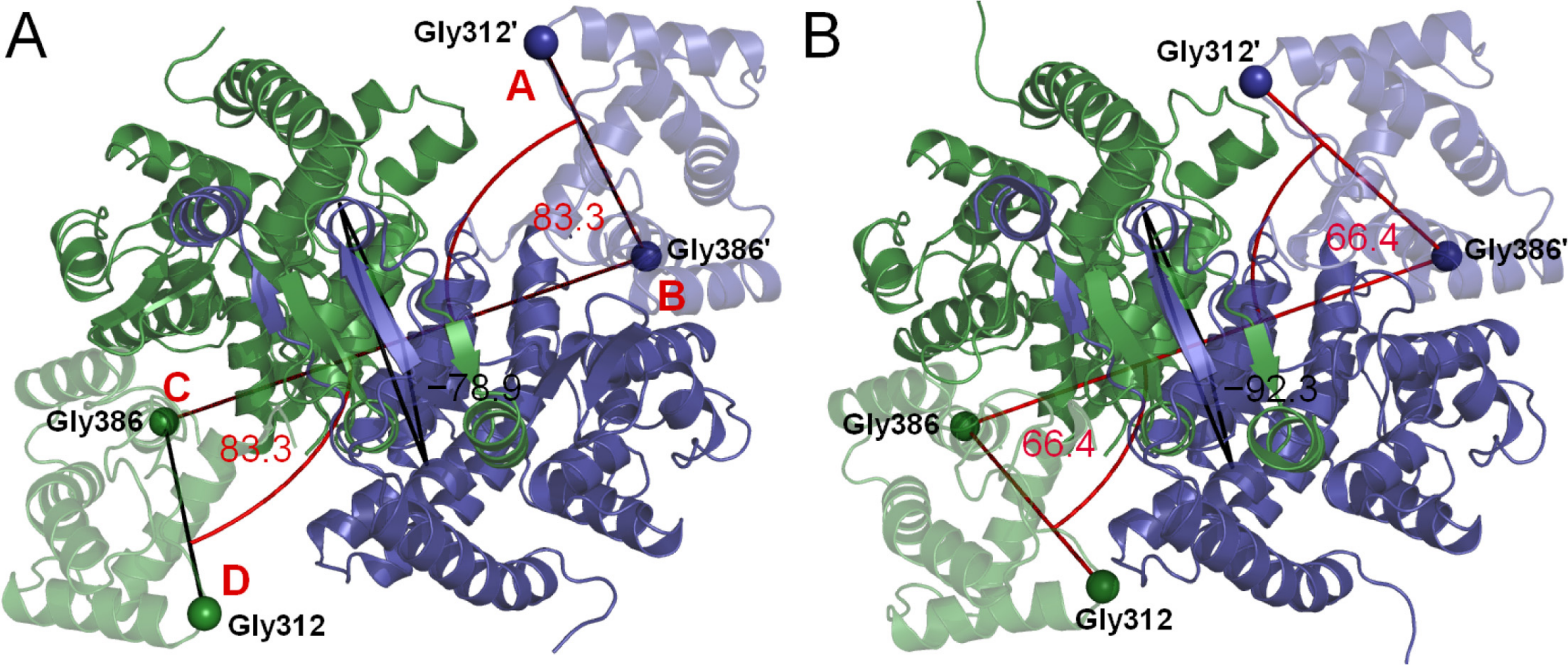
Pig heart citrate synthase (CS) crystal structures with the angle and dihedral measurements labelled. Chain A is depicted in dark blue, chain B in dark green, with the small domains transparent. A) open structure, PDB ID: 3ENJ.[23] B) closed structure, PDB ID: 2CTS.[7]

In the homodimeric citrate synthase (CS) structure, each chain contributes a large domain to the main body of the dimer, with a small domain connected to the main body by a flexible region[22]. This defines two active-site clefts in the dimer. Substrate binding in CS involves a substantial motion of the small domain to close the binding cleft. Crystallography gives evidence of doubly-open and doubly-closed (ligand bound) CS structures, apparently implying a cooperative functional motion in which the two clefts open or close together. Fig 4 shows both the open, unliganded structure of pig CS (3ENJ)[23] and a corresponding doubly liganded structure (2CTS)[7] obtained with the products citrate and coenzyme A bound. This symmetric closure of the two clefts is evident. Previous studies[9, 24] have noted that the vector describing this transition has a strong overlap with a normal mode of the CS structure, a property conserved in both mesophilic and extremophilic dimeric CS[12].

MD simulations of pig CS were performed starting from both the open (1CTS) and closed (2CTS) structures, as described in Methods. In each case, three independent 50ns trajectories were obtained. Geometric simulations of flexible motion were performed using the open (3ENJ) structure as input. Examination of the results of the coarse-grained elastic network modelling indicated that collective motions of the small domains relative to the main body of the dimer are described by the eigenvectors of the three lowest-frequency nontrivial normal modes–modes 7, 8 and 9. Higher modes are more localised. These lowest-frequency modes were therefore used to bias motion in the geometric simulations.

To visualise our results, we make use of an angular measure describing the attitude of the small domains relative to the main body of the CS dimer [12]. Selecting residues at the tips of the small domains and at the flexible "hinge" regions connecting them to the large domains, we take a succession of four residues in the order tip-hinge-hinge-tip (see Fig 4). This defines two cleft-closing angles ABC, BCD and a dihedral angle ABCD. We take Gly-312 as the tip of the small domain and Gly-386 as the hinge.

### Simulations of motion in CS

**Fig 5:**
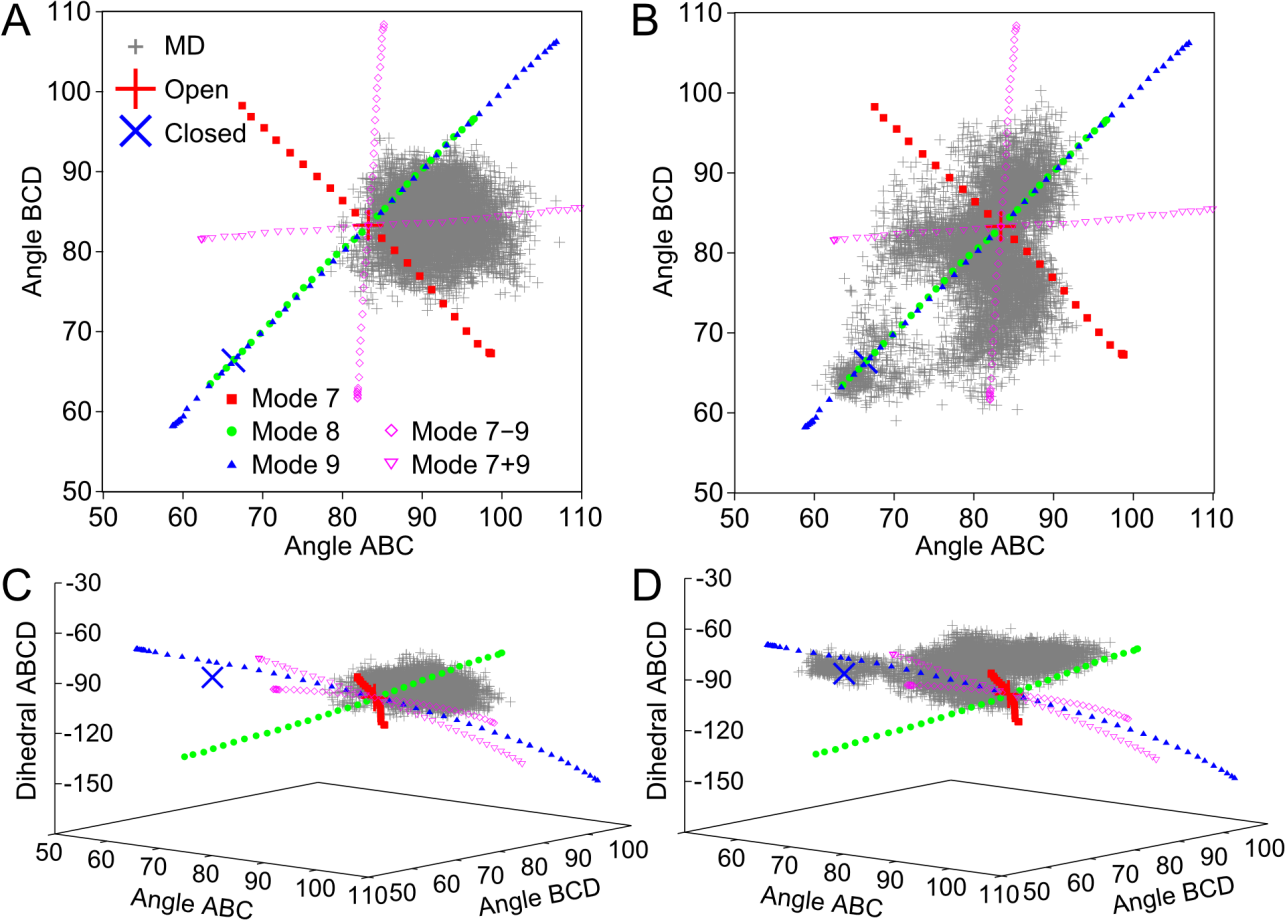
Comparison of geometric simulation and molecular dynamics trajectories of citrate synthase. Plots of the ABC and BCD angles (panels A,B) and ABCD dihedral (panels C,D) that define the active sites opening as observed in the flexible motion trajectories biased along modes 7, 8 and 9 (small closed symbols) and in the MD trajectories (plus signs for every 100 ps) obtained starting from the open structure (A,C) and the closed structure (B,D). Trajectories are also shown for motion biased along constructed modes “9+7” (open triangles) and “9–7” (open diamonds). Measurements for the open (PDB ID: 3ENJ) and closed (PDB ID: 2CTS) structures are also indicated. (For definitions of ABC and BCD angles and ABCD dihedral, see Fig 4.)

In Fig 5A and 5B we show data for motion starting from the open CS structure, both for MD and for geometric simulations biased in the direction of normal modes 7,8 and 9 and combinations thereof. Fig 5A shows the cleft-closing angles ABD, BCD, while Fig 5B shows the same data with the inclusion of the dihedral angle ABCD in a 3-D representation. It is clear that mode 7 is an anti-symmetric mode while modes 8 and 9 are symmetric modes which differ in the behaviour of the dihedral angle. Geometric simulations biased along mode 9 describe well the symmetric crystallographic open/closed transition between 3ENJ and 2CTS. This is fully consistent with earlier studies on normal modes of CS[9, 24]. By motion biased along this mode, the CS structure can become both significantly more open than the 3ENJ crystal structure, and slightly more closed than 2CTS, without violating the constraint network of the 3ENJ structure. As the geometric simulations use a simplified physical model this does not, of course, preclude the existence of substantial free energy barriers not represented in the local interactions defining the constraint network.

A striking feature of the anti-symmetric motion, meanwhile, is that such motion can apparently close one or other binding cleft to an extent comparable to that seen in the symmetrically closed structure. Since we expect the true dynamics of the structure to involve components of all the lower-frequency modes, this suggests that the structure will be able to display independent, non-cooperative motions in which one or other binding cleft opens and closes independently of the other. To test this proposition we have further carried out simulations of motion biased parallel and antiparallel to two constructed modes representing the sum and the difference of modes 9 and 7. Formally the bias eigenvectors are constructed as **e**_9+7_ = (1/sqrt(2))(**e**_9_ + **e**_7_) and as **e**_9-7_ = (1/sqrt(2))(**e**_9_—**e**_7_). Trajectories from geometric simulations of flexible motion biased using these modes are also shown on Fig 5A and 5B. These motions have the expected character, an effectively independent opening and closing motion of one cleft or the other as described by the angles ABC and BCD.

**Fig 6:**
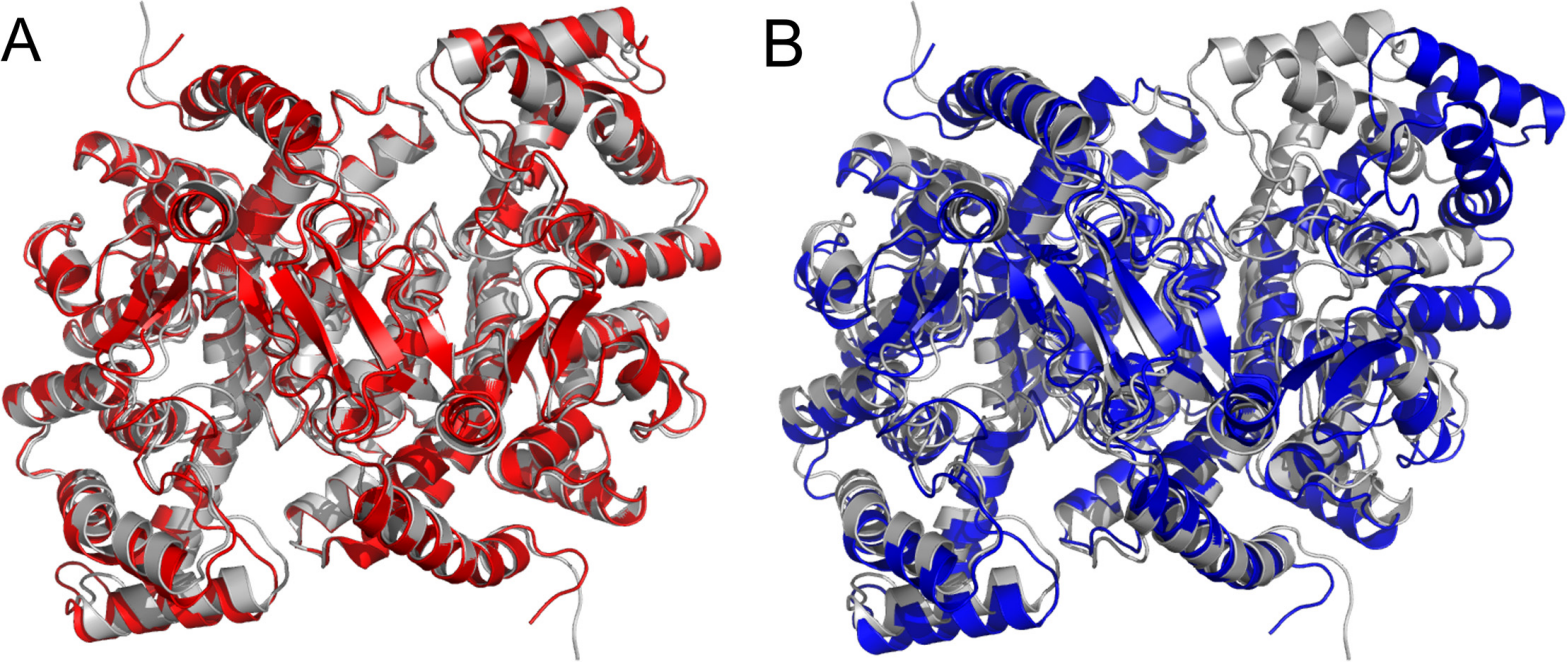
Symmetric (A) and asymmetric (B) forms of citrate synthase as generated by the geometric simulations (red, blue) biased along normal modes 9 (A) and 7 (B), started from the open structure (PDB ID: 3ENJ), compared to the closed structure (PDB ID: 2CTS, light gray).

Fig 6A shows an overlay of the closed (2CTS) structure with a frame from geometric simulations biased along normal mode 9 of 3ENJ. It is clear that the motion captures the character of the crystallographically observed symmetric transition. Fig 6B, meanwhile, shows an overlay of the closed (2CTS) structure with a frame from geometric simulations biased along normal mode 7 of 3ENJ. In this case the alignment is carried out only on the closing chain of the dimer, chain A. We can see that the simulated closure of the binding cleft in chain A in this anti-symmetric motion appears to match the closed cleft of the 2CTS structure just as well as the symmetric motion does. The other cleft, meanwhile, has opened significantly beyond what is seen in "open" crystal structures of CS. Motions biased along mode 8 do not generate cleft geometries relevant for the open-closed transition as they show a different behaviour of the dihedral angle describing torsion of the dimer.

The MD simulations starting with the open structure show several notable features. First of all, the conformational space explored (in terms of the cleft-closing angles) lies mostly further away from the closed structure. That is, the exploration of symmetric conformations–roughly equal change in both cleft angles compared to the starting structure–is towards structures where both clefts are more open rather than more closed. The range of motion explored by MD lies within the range defined by the jamming limits of the flexible motion simulations. The MD simulations do explore a considerable degree of closure in one of the chains (down to 72°), but this is achieved by noncooperative (independent) motion and not by cooperative symmetric motion; during this portion of the trajectories the correlation between the cleft angles is just below zero. Overall, the MD simulations indicate that the symmetric and the anti-symmetric motions suggested by the flexible motion simulations are approximately equally important for the dynamics of CS in solution (leading to largely non-cooperative motion), and that the solution structure is indeed capable of opening considerably further than the crystallographic open structure.

Fig 5C and 5D show data for atomistic MD simulations beginning from the closed (2CTS) unliganded structure. The flexible motion simulation trajectories from the open structure are duplicated here to provide a frame of reference for comparison. The range of motion observed in these trajectories is considerably greater than for the trajectories starting from the open form, as expected. Conformations in which both clefts are near the crystallographic closed structure are initially sampled. Over the course of the first 10ns of each simulation, however, both binding clefts open, and the bulk of the MD trajectory is visibly centred around the open rather than the closed conformation. Importantly, cleft opening does not happen simultaneously in both chains (i.e. not along the path of the symmetric modes identified), but rather one by one: in two runs, angle ABC opens up first, and in the other, the angle BCD opens up first (the angles are never simultaneously both around 73°). Further sampling of cleft movement appears to be predominantly independent rather than cooperative.

We can quantify the impression of non-cooperative motion in the MD simulations further by calculating the correlation between the angles ABC and BCD over moving windows during the course of each simulation. The first 10ns of each "from closed" simulation gives a strong positive correlation, as during this phase the two angles initially open. Over the remainder of the "from closed" simulations, however, and throughout the "from open" simulations, the correlations vary from somewhat positive to somewhat negative. When the first 10 ns of the trajectories are ignored (to allow for equilibration), the average correlation (*R*) and its standard deviation over all 6 simulations is −0.01 ± 0.20 for 5 ns windows and 0.01 ± 0.17 for 10 ns windows. Average values for “from open” simulations are similar to those for “from closed” simulations. This strongly indicates that the cleft motions of CS in solution are non-cooperative.

We have carried out a further analysis on the MD trajectories in order to validate the use of these angular measures to describe the domain motions. It might be objected that such measures are susceptible to error due to local fluctuations in the positions of the selected atoms. We have therefore divided the MD trajectories into 1ns windows and calculated the standard deviation of variations in the angular measures within each window. The resulting standard deviations lie between 1.2 and 2.8 degrees for 95% of the windows, extending up to 4.0 degrees for 5% of outliers where the window includes a more substantial domain motion. Comparison of the cleft-closing angles ABC,BCD and the dihedral ABCD with RMSD measures of the change of each of the small domains (with respect to the main body of the dimer) upon opening indicates that these measures adequately track the conformational change (S7 Fig). Clustering of the “from closed” MD trajectories on this RMSD measure was also performed, indicating that the resulting cluster centroids are well distributed over the range covered by the cleft-closing angles (S8 Fig).

Our current MD simulations confirm the main conclusions from previous conventional[10] and enhanced sampling[11] MD simulations at much shorter timescales: in the absence of ligands, the closed conformation opens rapidly, and open conformations do not close spontaneously. This is consistent with a picture in which the closed apo-conformation has a significantly higher free energy than the open apo-conformation. The “from open” MD simulations further indicate the existence of a ‘nearly open’ state, which may be similar to the state reached in the enhanced sampling simulations that targeted the open structure from the closed. According to PCA analysis of our trajectories (see S3 Fig), the conformations sampled by two (out of six) chains in “from closed” simulations coincide with those sampled in the “from open” simulations, indicating that a full transition to the “open” ensemble in solution is reached. This indicates that any barrier between the open and closed (as suggested previously[11]) should be reasonably low, as 50 ns unbiased MD simulation at 310 K can overcome it.

This example thus brings out the value of applying appropriate modelling techniques to obtain information that is implicit in the crystal structure of proteins. In this case, the anti-symmetric cooperative motion cannot be inferred from comparison of crystal structures, whereas the symmetric cooperative motion can; this is because the crystallisation process will tend to obtain either a double-open or a double-closed, ligand-bound conformation.

## Discussion

### Biological context of DcpS and citrate synthase function

The geometric simulations and the MD simulations performed here describe the functional motions in two different domain-swapped homo-dimeric enzymes. In DcpS, we find a clear asymmetric cooperative motion; in the absence of ligand, a near-closed conformation can be attained in one active site, while the other active site is open–or vice-versa. Substrate binding is thus probably largely governed by conformational selection of the unliganded enzyme, with further subtle rearrangements to allow for reactive alignment with the catalytic histidine triad. Further, the flexible motion and MD simulations confirm that simultaneous closure of both active sites is structurally and energetically impossible. It appears that the dimer structure has been naturally selected to enhance cooperative motions, probably to allow efficient substrate binding and product release in what is essentially half-of-sites reactivity;[25] the negative cooperativity in the dimeric enzyme thus enhances enzyme activity (under conditions of low substrate concentration). In CS, essentially no cooperative motion occurs in the unliganded enzyme, which strongly prefers open conformations of both active sites. It is well known that the two substrates, oxaloacetate and acetyl-CoA, bind in an ordered sequential manner (under the low acetyl-CoA concentrations typically observed in vivo):[26, 27] oxaloacetate binds first, followed by acetyl-CoA. Given that the unliganded enzyme prefers the open conformation, the mechanism of oxaloacetate binding is believed to be predominantly induced fit[27]. The conformational change in response to oxaloacetate binding (and subsequent acetyl-CoA binding) leads to a well-organized active site, in which both substrates are sequestered from solvent. This sequestration is functionally important, as it helps avoid premature hydrolysis of acetyl-CoA (and potentially also decarboxylation of oxaloacetate), which would stop citric acid cycle turnover. The well-organized closed state also offers a means to couple chemical and conformational change[28]. Once condensation has taken place to form citryl-CoA, a more open conformation of the active site is required to allow the next chemical step catalyzed by CS: hydrolysis of citryl-CoA to form citrate and CoA. Closure and re-opening are thus functionally important, but cooperative closure of both binding sites upon oxaloacetate binding to one site is not desired; this would potentially slow down the citric acid cycle, by preventing catalysis in the second active site. Citrate synthase is also known to associate physically with other enzymes such as malate dehydrogenase[29, 30] and pyruvate dehydrogenase[31]; the capacity for independent action of the two binding sites may be relevant to maintaining function under these conditions.

The two contrasting examples of conformational change in the homodimeric enzymes described here illustrate that the commonly observed formation of domain-swapped homodimers can have distinctly different implications for their conformational dynamics, which are likely to have evolved to serve different purposes[32, 33]. The conformational dynamics are encoded in the structure, and provide a clear reason for selection of domain-swapped homodimers by evolution. It is also clear that different domain-swapped homodimeric enzymes can show significantly different dynamics. Molecular simulations provide an excellent route to explore and analyse such functionally relevant motions.

The combination of flexible motion simulation and conventional MD simulation is powerful: the former allows the rapid exploration of structurally feasible conformational transitions and the latter indicates which regions of the conformational landscape are physically and energetically feasible.

## Conclusions

Conformational changes are an integral aspect of catalytic cycles for many enzymes. Molecular simulations have an essential role to play in establishing structural and kinetic details of conformational changes. We find a remarkable degree of agreement between two modelling approaches using very different levels of theory–conventional empirical-potential atomistic MD, and rapid geometric simulations of flexible motion–in exploring the motion of two very different dimeric enzyme structures whose function involves large-scale domain motion. This agreement does not, of course, mean that the methods generate directly comparable dynamic trajectories, due to the very different levels of theory which they employ. Rather, we find that the methods concur as to the character of the large-scale motion which is an intrinsic property of the open crystal structures, the computational exploration of which provides information beyond what can be inferred from comparison of the static open and closed crystal structures. The rapid geometric simulation method employed here has shown considerable utility in modelling protein flexibility for the interpretation of experimental data; this direct comparison with conventional MD provides a useful cross-validation of the approach. We note that geometric simulations using FRODA without normal mode information, and elastic network modelling without geometric simulation, have previously been compared with MD [34] with promising results.

In DcpS, where crystallography identifies a potential anti-symmetric cooperative functional motion, we identify the motion involved as an intrinsic feature of the enzyme structure. We also identify a symmetric cooperative motion, with limited amplitude, occurring when the structure is in the near-symmetric, doubly-open state. MD simulations indicate that the anti-symmetric motion dominates and both asymmetric conformations are accessible without a significant free energy barrier. The two simulation methods concur that components of the symmetric motion are also involved in the full translation from a structure closed on one side to a structure closed on the other. In CS, where crystallography identifies a potential symmetric cooperative functional motion, we identify symmetric cooperative, anti-symmetric cooperative, and non-cooperative motions as intrinsic features of the enzyme structure. The geometric simulations identify the anti-symmetric cooperative motion as being potentially as important as the symmetric motion, and thus assist in the interpretation of the MD results. The MD simulations give further insight into the range of conformations along these motions that can be accessed, and indicate that physically realistic protein motions are essentially non-cooperative. The agreement between the two modelling approaches strongly implies that non-cooperative motions are just as much an intrinsic feature of the CS structure as the symmetric cooperative motion implied by crystallography. DcpS and CS thus represent two contrasting examples of homodimeric enzymes. DcpS displays a conformational selection mechanism of ligand binding, and different simulation methods both indicate a large anti-symmetric cooperative motion in the open-to-closed transition (as suggested by crystallography). CS is believed to display an induced fit mechanism[27] of ligand binding, and geometric and MD simulation together identify non-cooperative behaviour of the dimeric structure (whereas crystallography appears to suggests a symmetric cooperative open-to-closed transition). The two examples illustrate the value of applying rapid, inexpensive geometric simulations of flexible motion in conjunction with standard MD simulation: in cases where large domain motions are expected, we recommend the use of both methods together to gain insight into the nature and mechanism of conformational change.

## Methods

### Explicit-solvent molecular dynamics simulations

For DcpS, four independent trajectories of 100 ns each were performed, starting from the approximately symmetric apo-form, based on the crystal structure with PDB ID 1XML.[1] The preparation of the starting structure, equilibration and production simulation were done as described previously[3]; we provide only a short description here. Missing loops were modelled in and mutations introduced for crystallization purposes (Leu206Met, Leu317Met) were changed back to the wild type Leu. In addition to the water molecules from the X-ray structure, a rectangular box of water extending at least 13 Å from the protein was added, as well as seven sodium ions to neutralize the system. Minimization and MD simulation was then performed using NAMD 2.6[35], with the CHARMM27 protein force field[36] and the TIP3P water model[23, 37] and a 2 fs time-step (with SHAKE[38] applied to bonds involving hydrogens). A 12 Å cutoff was used for van der Waals and short-range electrostatic interactions, and PME for long-range electrostatics. Heating and 1 ns of equilibration was performed, first in the NVT, then the NPT ensemble. Production simulations were conducted in the NPT ensemble, keeping the temperature at 300 K using Langevin dynamics and the pressure to 1 atm using a Nosé-Hoover Langevin piston.

For CS, 6 independent simulations of 50 ns each were performed, 3 starting from the open form (PDB ID: 1CTS) and 3 from the closed form (with coenzyme A and citrate removed; PDB ID: 2CTS) of the pig (*Sus scrofa*) enzyme. Dimers were constructed using crystallographic symmetry and hydrogens were added using the CHARMM program[39] v. 30. The histidine residues in the active site were treated as singly protonated on Nδ (His235 and His274) or Nε (His238 and His320), in line with their local hydrogen-bonding environments and similar to treatment in previous computational studies (double protonation leads to active site distortion[40, 41]). Other protonation states and histidine tautomers were determined from the optimal hydrogen bonding network[42] calculated with the WHAT-IF web-interface (http://swift.cmbi.ru.nl). The structures were solvated in water as described above, and sodium and chloride ions were added such that the net charge of the system was zero and the average ionic concentration of the solution was 0.05 mol dm^−3^. Solvent positions were equilibrated and subsequently the whole system was heated and equilibrated prior to production simulation in the NPT ensemble at 310 K (close to the average pig body temperature of ~312K) and 1 atm, with temperature and pressure regulated as above, again using the NAMD software with the CHARMM27 protein force field[36], the TIP3P water model[23, 37] and a 2 fs timestep (combined with SHAKE[38]).

### Rapid geometric simulations of flexible motion

This approach[20] combines information from rigidity analysis and coarse-grained elastic network normal mode analysis to explore large amplitudes of motion in an all-atom model, using template-based geometric simulation to maintain the bonding geometry and the network of noncovalent interactions identified in the input structure. We use Elnemo software[18] to obtain normal mode eigenvectors from coarse-grained elastic network modeling, and FIRST/FRODA software[17, 19] to carry out rigidity analysis (FIRST)[43], identifying the noncovalent interaction network and labelling dihedral angles as locked or variable, and template-based geometric simulations of flexible motion (FRODA)[19] which project the all-atom structure over large amplitudes of motion while maintaining local bonding and steric geometry. The approach neglects all long-range interactions, and thus sacrifices information about the detailed conformational energy landscape, in favour of the ability to explore physically plausible motions in an all-atom structural model over large length scales at minimal computational cost[20, 44, 45]. An exploration of motion along multiple normal mode directions, covering substantial (multi-Ångstrom) motions of a protein with several hundred residues, can typically be completed with a computational expense of a few CPU-hours. Recent studies[46, 47] have shown that, despite the simplifications involved in the model, flexible motion simulations are informative for protein structural biology and biochemistry. Normal-mode-based geometric simulations have identified a cisplatin-binding mechanism in calmodulin[47], domain motions carried out by ERp27 in solution[46], and flexible variations of the target in protein folding simulations[48]. In a recent study, investigations of rigidity and flexibility in newly solved structures of mutants of calexcitin were reported alongside the crystal structures themselves[49]. However, no direct comparison with MD has previously been published.

We generate normal mode eigenvectors in Elnemo in a one-site-per-residue coarse-graining using the Cα geometry of the input structure, placing springs of equal spring constant between all sites lying within an interaction distance cutoff of 12 Å. For a protein of *N* residues this generates a 3*N*x3*N* matrix of second derivatives; diagonalisation generates a set of 6 trivial zero-frequency modes, representing combinations of rigid-body rotations and translations of the input structure, and 3*N*-6 nontrivial modes. When sorted in ascending order of frequency, the lowest-frequency nontrivial mode is thus mode 7. Since the lowest-frequency nontrivial modes represent directions along which motion can occur with little restoring force, we expect these low-numbered modes to contribute strongly to the large-amplitude flexible motion of the structure and to form a "basis set" describing flexible motion.

A rigidity analysis of the all-atom input structure is carried out in FIRST using the "pebble game" algorithm[17, 50] which matches degrees of freedom against bonding constraints in the molecular framework of the protein. Bonding constraints include covalent, hydrophobic and polar (hydrogen bond and salt bridge) interactions. As the strength of the polar interactions can be gauged from their geometry, the results of the analysis depend on an "energy cutoff" which selects the set of polar interactions to include in the constraint network[43]. Previous research has shown that, while interesting features of the static rigidity appear as the cutoff is varied around the range[43, 51] of −0.5 to −1.0 kcal/mol (for comparison, *R*T at room temperature is about 0.6 kcal/mol), flexible mobility is best explored at lower cutoffs around −2.0 to −3.0 kcal/mol[46, 47]. A cutoff of -2.0 kcal/mol is used in this study for both DcpS and CS.

Template-based geometric simulation of flexible motion, carried out using FRODA, explores the mobility of the all-atom structure using an iterative approach. In each step of the simulation, all atomic positions are slightly perturbed along the direction of a normal-mode eigenvector (a typical perturbation step being of order 0.01 Å along the bias direction, along with a random step also of order 0.01 Å), followed by a relaxation of the atomic positions to satisfy steric exclusion and maintain bonding geometry (represented as a series of overlapping multi-atom templates). All longer range interactions are neglected. For each mode of interest we explore motion biased parallel and antiparallel to the normal mode eigenvector and carry out several thousand iteration steps to generate large motion amplitudes. The simulation generates an initial phase of "easy" motion, where the bonding geometry is easily maintained[20], followed by the onset of "jamming" as the motion encounters steric and bonding constraints which naturally limit its amplitude.

### Analysis

Distance, angle and dihedral measurements and principal component analysis (PCA) of MD trajectories were performed using WORDOM[52]. PCA for DcpS was performed by combining the four trajectories after alignment on the Cα atoms of the C-terminal domains. (The final 60 ns of run 4 was omitted for PCA, as the dimer structure remains ‘stuck’ in a single, asymmetric, conformation.) Subsequently, the projection of the individual trajectories onto the first two PCA eigenvectors was calculated. Pearson correlations between the distance measurements in DcpS and the angle measurements in CS (that define how open the active sites are, see Figs 1 and 4) were calculated using R (www.r-project.org), either over the whole trajectory or for moving ‘windows’; in the latter case, correlation was calculated as a function of simulation time. All molecular figures were prepared with PyMOL[53] (www.pymol.org).

## Supporting Information

S1 Fig Comparison of flexible motion and molecular dynamics trajectories of DcpS. Plots of the angle between Cα-atoms of Tyr143-Arg145-Arg149 in chain A and B of DcpS as observed in the flexible motion trajectories along modes 7, 8 and the linear combination of 7+8 (small closed symbols) and in the individual MD trajectories (plus signs for every 100 ps); A) run 1, B) run 2, C) run 3, D) run 4.
(TIF)

S2 Fig Projections of the four individual DcpS MD trajectories on the first two eigenvectors determined by principal components analysis. The (symmetric) starting structure (1) and structures most similar (according to Ca RMSD) to the asymmetric 1XMM (2) or 1XMM-chain swapped (3) structures are also indicated.
(TIF)

S3 Fig Validation of distance measures for DcpS using clustering. Distribution of representative structures of clusters (cluster centroids, open red circles) obtained from the full MD trajectories of DcpS, identified based on RMSD, in the space of the d(AB)/d(BA) variables. Grey points are MD frames from all four trajectories; filled circle is input structure; closed and open squares are closed structures. Cluster centroids are well spaced across the range described by the d(AB)/d(BA) measures. Hierarchical agglomerative clustering was performed on the Cα RMSD of the N-terminal domains, after fitting to the Cα RMSD of the C-terminal domains (using cpptraj from AmberTools14). The average distance to centroid of the 20 target clusters ranges from 0.83 to 1.85 Å.
(TIF)

S4 Fig Validation of single intersubunit distance measures to describe cleft opening and closing in DcpS. Correlation between the single cleft distance measure (d(AB)/d(BA), between Cα Asp111 to Cα Trp175’, as used in the main text) and the distance root-mean-square of six different cleft distances (dRMS) in the course of the MD trajectories for DcpS. The six cleft distances are the intersubunit distances of Cα’s of Asn110 and Thr130 (on the N-terminal domains) to Cα’s of Trp175’, His279’, Asp205’ (on the C-terminal domains). Each row is an MD trajectory. Left column, measures d(AB) and d(BA); right column, dRMS measures. The single distance measure clearly captures the cleft opening of each trajectory.
(PNG)

S5 Fig Flexible motion trajectories compared to MD PCA eigenvectors. Projection of the flexible motion trajectories for DcpS biased along modes 7,8 and 7+8, onto the PC1,2 bases obtained for DcpS from different sections of the MD trajectory. Colours show the projections onto PC1,2 for a full trajectory 0-100ns (A) (black) and for the subsets 0-75ns (J) (red), 0-85ns (K) (yellow) and 10-100ns (L) (blue). This visually confirms the findings from S1 Table that PC1,2(A) and PC1,2(L) are effectively identical, while PC1,2(J) and PC1,2(K) are rotations of the basis vectors in the same space.
(PNG)

S6 Fig Comparison of angle and dihedral variables to RMSD for CS. Figures show a comparison of the angle and dihedral variables for CS (as described in the main text) to the Cα RMSD of the small domains (residues 275–385; red and blue in panel A) after alignment on the stable part of the large-domain dimer (residues 5–54, 89–270 and 390–436; black in panel A). Both measures describe motion of the small domains in the course of the first 20ns of the 3 “from closed” MD trajectories (where the closed-to-open transition occurs; see main text). In panels B-D, red and blue lines show RMSD of the individual small domains, orange and cyan lines the equivalent angle measures, grey line the dihedral measure and black the Cα RMSD of the stable large-domain dimer. In two cases (panels B,C) the RMSD and the cleft-opening angle measures track each other closely; in the third case (panel D), for one of the domains (blue), the RMSD and cleft opening angle diverge, due to variation in the dihedral (domain rotation) angle. Overall, these data show that the angle and dihedral variables well capture the large scale motion of the small domains relative to the main body of the CS dimer.
(PNG)

S7 Fig Validation of angle and dihedral measures using clustering. Distribution of representative structures of clusters (cluster centroids, open red circles) obtained from the full ‘from closed’ MD trajectories of CS, in the space of the angle and dihedral variables. The RMSD clusters are well spaced over the range of motion explored by the MD in the space of the angular variables. Grey points are MD frames from all four trajectories; filled circle is input structure; closed and open squares are closed structures. Hierarchical agglomerative clustering was performed on the Cα RMSD of the small domains (red or blue in S6A Fig), after fitting to the Cα RMSD of the large domain dimer (black in S6A Fig), using cpptraj from AmberTools14. The average distance to centroid of the clusters obtained from each subunit (10 clusters each) ranges from 1.10 to 1.46 Å.
(TIF)

S8 Fig Projections of the combined ‘from open’ and ‘from closed’ trajectories of citrate synthase onto the first two eigenvectors determined by principal component analysis. A) the three combined trajectories for the ‘from open’ (black and blue lines show the projections for subunit A and B respectively) and ‘from closed’ trajectories (red and green lines show the projections for subunit A and B respectively; Xs mark the positions of the closed monomers A and B, as indicated). B-D) the ‘from closed’ trajectories broken down into the individual simulations (red and green lines for subunit A and B respectively with the space filled in brown for the ‘from open’ comparison set).
(TIF)

S1 Table Generalised dot products among the principal component eigenvectors PC1/PC2 obtained over different sections of an MD trajectory for DcpS. PC1,2 for a full trajectory 0-100ns (A) are compared to those for the subsets 0-75ns (J), 0-85ns(K) and 10-100ns (L). The dot products show that sets A and L are effectively identical. For sets J,K compared to A, there is some mode mixing; however the sum of squares data show that PC1,2(J and K) cover the same space as PC1,2(A) and are simply rotations of the same basis vectors.All data are given to three significant figures and derived from eigenvectors at five significant figures. Each PC eigenvector consists of three components (x,y,z) for the motion of each residue in the structure. The generalised dot product is formed simply by summing the products of corresponding entries in two eigenvectors.
(PDF)
