# Supplementary figures and images for: Structure and Function in Homodimeric Enzymes: Simulations of Cooperative and Independent Functional Motions
Source: PLoS One. 2015 Aug 4;10(8):e0133372. doi: 10.1371/journal.pone.0133372 (PMC4524684; doi:10.1371/journal.pone.0133372)

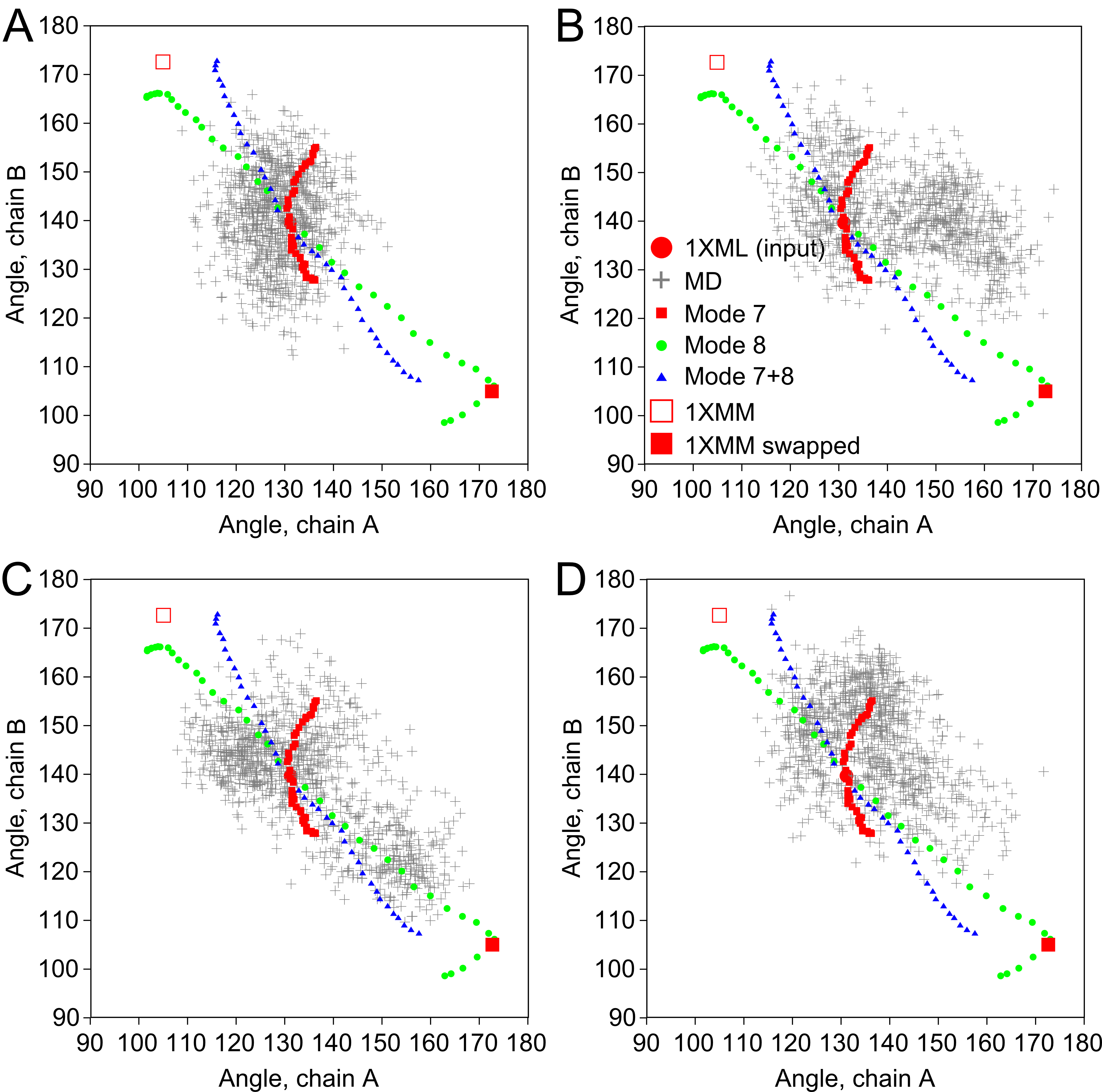

Supplement: S1 Fig — Plots of the angle between Cα-atoms of Tyr143-Arg145-Arg149 in chain A and B of DcpS as observed in the flexible motion trajectories along modes 7, 8 and the linear combination of 7+8 (small closed symbols) and in the individual MD trajectories (plus signs for every 100 ps); A) run 1, B) run 2, C) run 3, D) run 4. (TIF) [file pone.0133372.s001.tif]

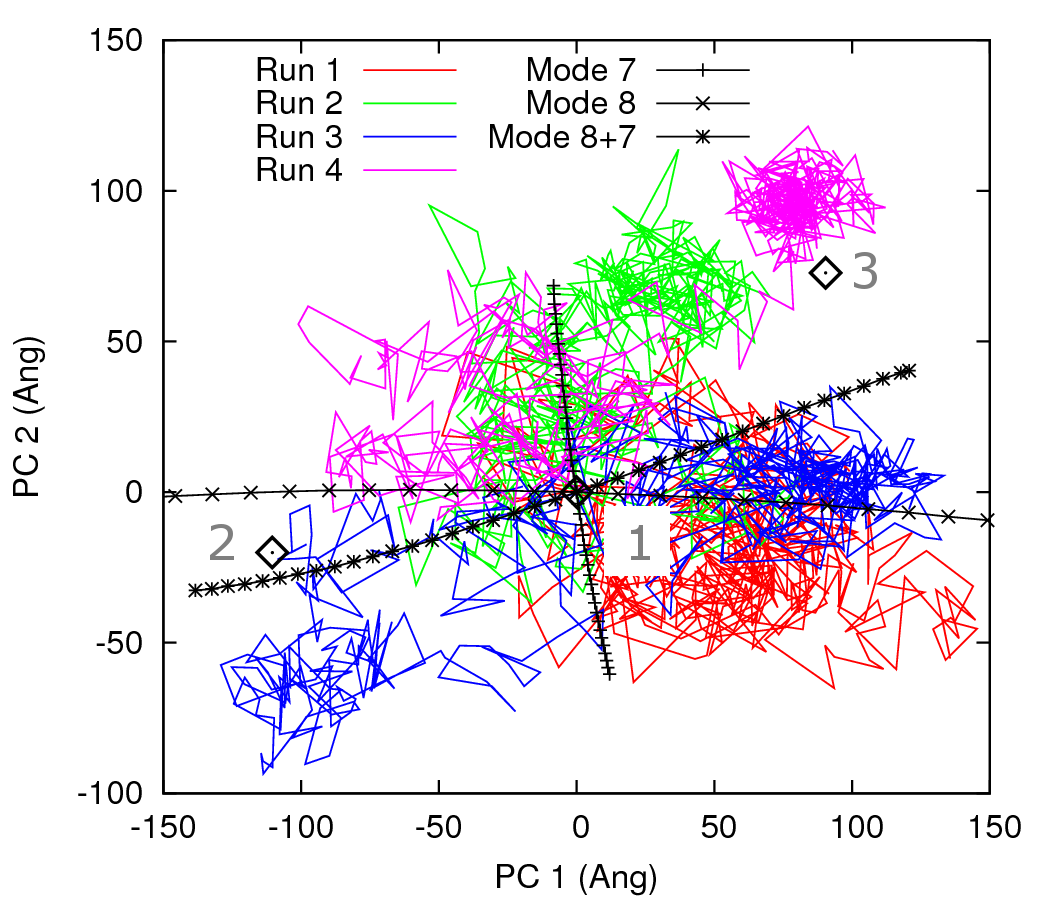

Supplement: S2 Fig — The (symmetric) starting structure (1) and structures most similar (according to Ca RMSD) to the asymmetric 1XMM (2) or 1XMM-chain swapped (3) structures are also indicated. (TIF) [file pone.0133372.s002.tif]

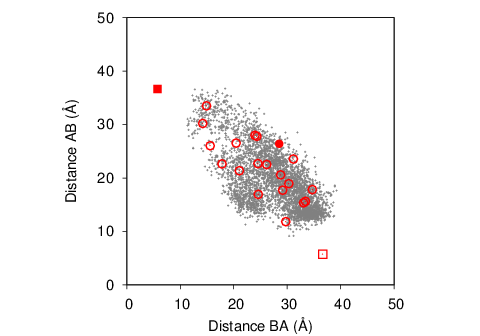

Supplement: S3 Fig — Distribution of representative structures of clusters (cluster centroids, open red circles) obtained from the full MD trajectories of DcpS, identified based on RMSD, in the space of the d(AB)/d(BA) variables. Grey points are MD frames from all four trajectories; filled circle is input structure; closed and open squares are closed structures. Cluster centroids are well spaced across the range described by the d(AB)/d(BA) measures. Hierarchical agglomerative clustering was performed on the Cα RMSD of the N-terminal domains, after fitting to the Cα RMSD of the C-terminal domains (using cpptraj from AmberTools14). The average distance to centroid of the 20 target clusters ranges from 0.83 to 1.85 Å. (TIF) [file pone.0133372.s003.tif]

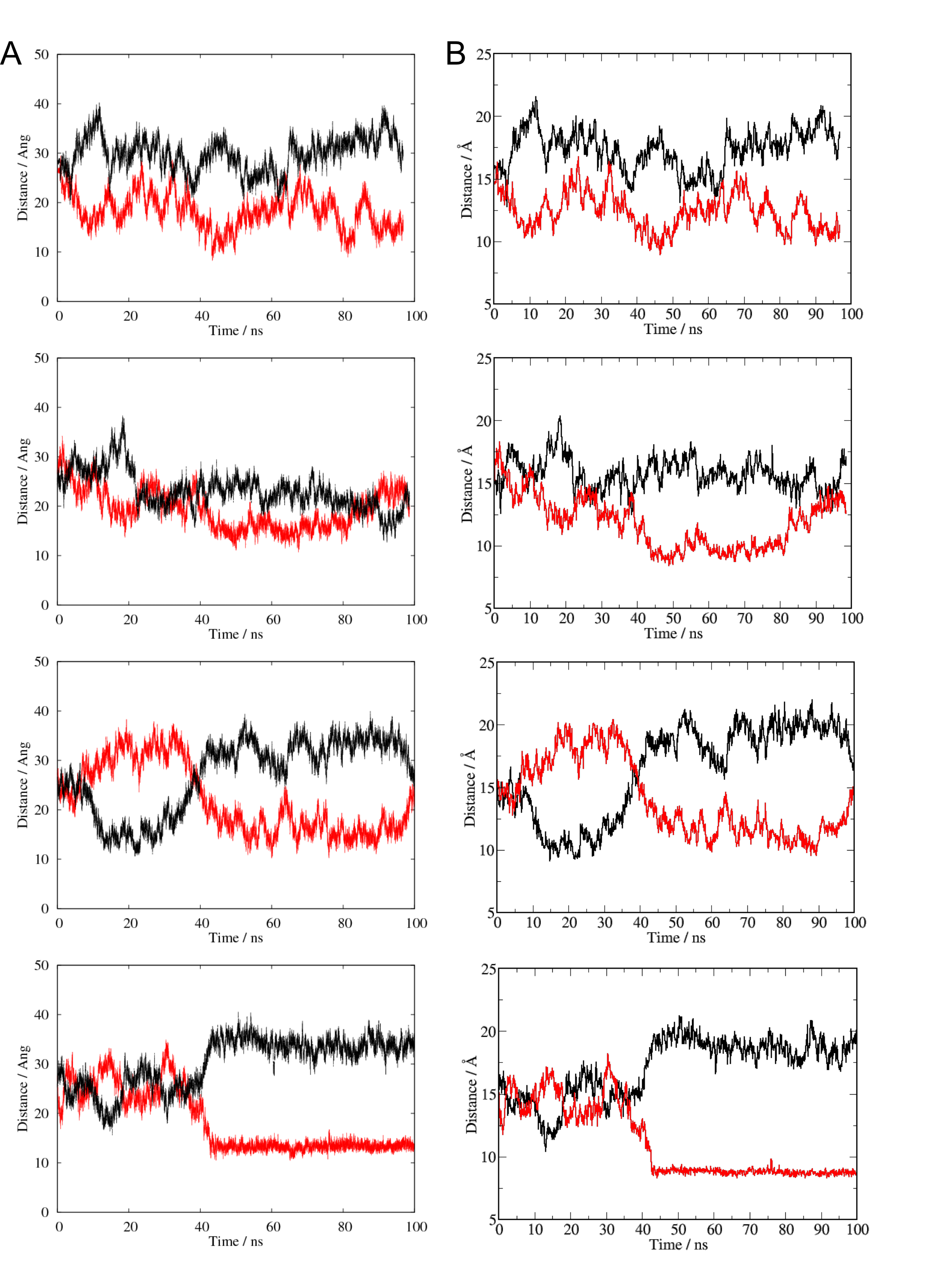

Supplement: S4 Fig — Correlation between the single cleft distance measure (d(AB)/d(BA), between Cα Asp111 to Cα Trp175’, as used in the main text) and the distance root-mean-square of six different cleft distances (dRMS) in the course of the MD trajectories for DcpS. The six cleft distances are the intersubunit distances of Cα’s of Asn110 and Thr130 (on the N-terminal domains) to Cα’s of Trp175’, His279’, Asp205’ (on the C-terminal domains). Each row is an MD trajectory. Left column, measures d(AB) and d(BA); right column, dRMS measures. The single distance measure clearly captures the cleft opening of each trajectory. (PNG) [file pone.0133372.s004.png]

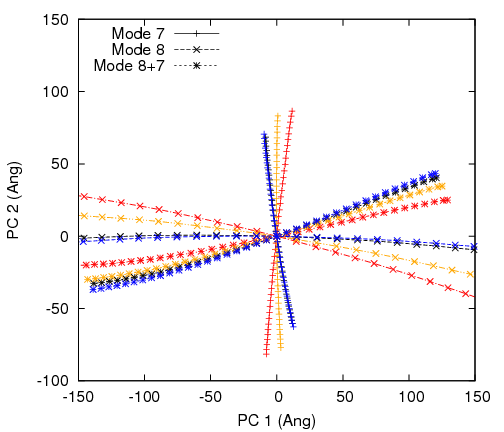

Supplement: S5 Fig — Projection of the flexible motion trajectories for DcpS biased along modes 7,8 and 7+8, onto the PC1,2 bases obtained for DcpS from different sections of the MD trajectory. Colours show the projections onto PC1,2 for a full trajectory 0-100ns (A) (black) and for the subsets 0-75ns (J) (red), 0-85ns (K) (yellow) and 10-100ns (L) (blue). This visually confirms the findings from S1 Table that PC1,2(A) and PC1,2(L) are effectively identical, while PC1,2(J) and PC1,2(K) are rotations of the basis vectors in the same space. (PNG) [file pone.0133372.s005.png]

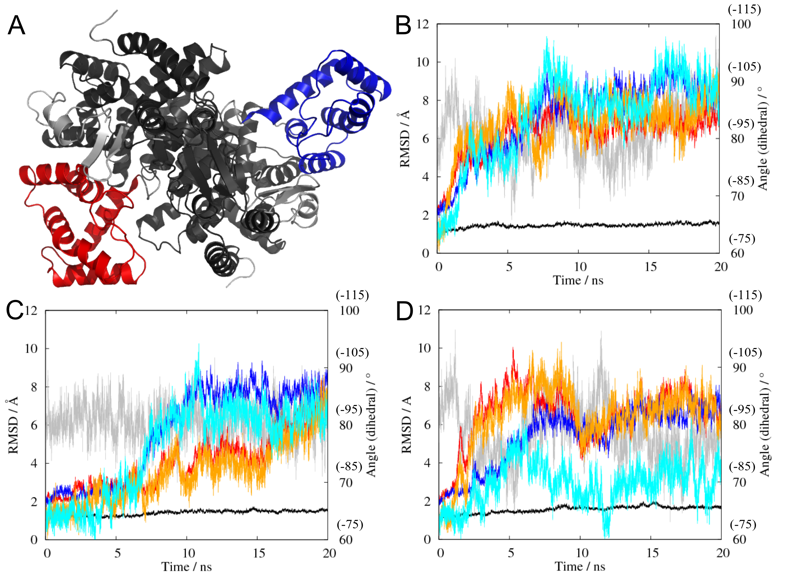

Supplement: S6 Fig — Figures show a comparison of the angle and dihedral variables for CS (as described in the main text) to the Cα RMSD of the small domains (residues 275–385; red and blue in panel A) after alignment on the stable part of the large-domain dimer (residues 5–54, 89–270 and 390–436; black in panel A). Both measures describe motion of the small domains in the course of the first 20ns of the 3 “from closed” MD trajectories (where the closed-to-open transition occurs; see main text). In panels B-D, red and blue lines show RMSD of the individual small domains, orange and cyan lines the equivalent angle measures, grey line the dihedral measure and black the Cα RMSD of the stable large-domain dimer. In two cases (panels B,C) the RMSD and the cleft-opening angle measures track each other closely; in the third case (panel D), for one of the domains (blue), the RMSD and cleft opening angle diverge, due to variation in the dihedral (domain rotation) angle. Overall, these data show that the angle and dihedral variables well capture the large scale motion of the small domains relative to the main body of the CS dimer. (PNG) [file pone.0133372.s006.png]

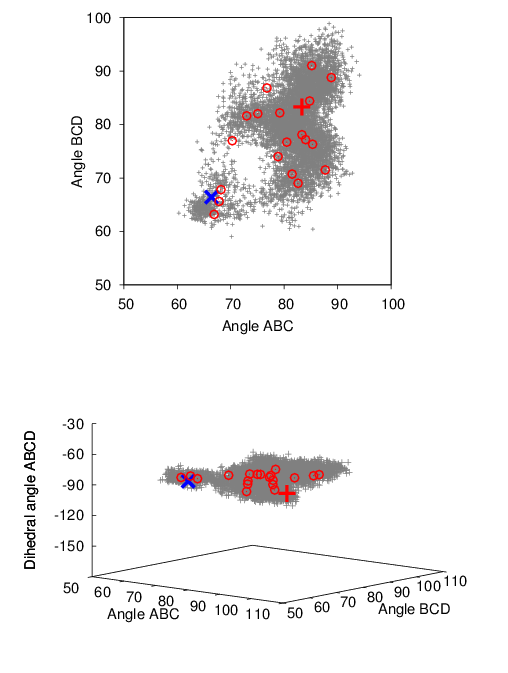

Supplement: S7 Fig — Distribution of representative structures of clusters (cluster centroids, open red circles) obtained from the full ‘from closed’ MD trajectories of CS, in the space of the angle and dihedral variables. The RMSD clusters are well spaced over the range of motion explored by the MD in the space of the angular variables. Grey points are MD frames from all four trajectories; filled circle is input structure; closed and open squares are closed structures. Hierarchical agglomerative clustering was performed on the Cα RMSD of the small domains (red or blue in S6A Fig), after fitting to the Cα RMSD of the large domain dimer (black in S6A Fig), using cpptraj from AmberTools14. The average distance to centroid of the clusters obtained from each subunit (10 clusters each) ranges from 1.10 to 1.46 Å. (TIF) [file pone.0133372.s007.tif]

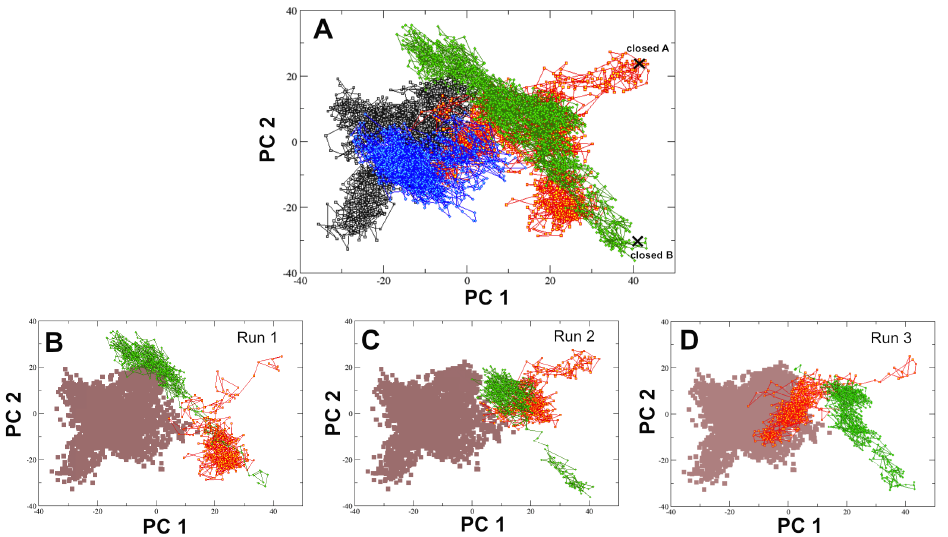

Supplement: S8 Fig — A) the three combined trajectories for the ‘from open’ (black and blue lines show the projections for subunit A and B respectively) and ‘from closed’ trajectories (red and green lines show the projections for subunit A and B respectively; Xs mark the positions of the closed monomers A and B, as indicated). B-D) the ‘from closed’ trajectories broken down into the individual simulations (red and green lines for subunit A and B respectively with the space filled in brown for the ‘from open’ comparison set). (TIF) [file pone.0133372.s008.tif]
