# Supplementary material for: Structure and Function in Homodimeric Enzymes: Simulations of Cooperative and Independent Functional Motions
Source: PLoS One. 2015 Aug 4;10(8):e0133372. doi: 10.1371/journal.pone.0133372 (PMC4524684; doi:10.1371/journal.pone.0133372)
Supplement: S1 Table — PC1,2 for a full trajectory 0-100ns (A) are compared to those for the subsets 0-75ns (J), 0-85ns(K) and 10-100ns (L). The dot products show that sets A and L are effectively identical. For sets J,K compared to A, there is some mode mixing; however the sum of squares data show that PC1,2(J and K) cover the same space as PC1,2(A) and are simply rotations of the same basis vectors.All data are given to three significant figures and derived from eigenvectors at five significant figures. Each PC eigenvector consists of three components (x,y,z) for the motion of each residue in the structure. The generalised dot product is formed simply by summing the products of corresponding entries in two eigenvectors. (PDF) [file pone.0133372.s009.pdf]

Dot…	With…							
	PC1(A)	PC2(A)	PC1(J)	PC2(J)	PC1(K)	PC2(K)	PC1(L)	PC2(L)
PC1(A)	1.000	0.000	0.968	-0.237	0.990	-0.135	1.000	0.012
PC2(A)	0.000	1.000	0.232	0.956	0.136	0.983	-0.012	1.000
Sum of squares: (PCn.PC1')^2 + (PCn.PC2')^2								
PC1(A)	1.000		0.993		0.998		1.000	
PC2(A)	1.000		0.967		0.984		1.000	
